# Snakebites in Cameroon: Tolerance of a Snake Antivenom (Inoserp™ PAN-AFRICA) in Africa in Real-Life Conditions

**DOI:** 10.3390/toxins16040165

**Published:** 2024-03-22

**Authors:** David Benhammou, Jean-Philippe Chippaux, Rodrigue Ntone, Yoann Madec, Pierre Amta, Gaëlle Noel, Fai Njuwa Karl, Anaïs Perilhou, Lucrece Matchim, Marie Sanchez, Mark Ndifon, Pedro Clauteaux, Lucrèce Eteki, Yap Boum, Armand Seraphin Nkwescheu, Fabien Taieb

**Affiliations:** 1 Emerging Diseases Epidemiology Unit, Institut Pasteur, Paris Cité University, F-75015 Paris, France; davidbenhammou@outlook.com (D.B.);; 2 MERIT Unit, Institut de Recherche pour le Développement, Paris Cité University, F-75006 Paris, France; 3 Epicentre Yaounde, Yaounde BP 12069, Cameroon; rodriguentone@hotmail.com (R.N.); karlnjuwagwei@gmail.com (F.N.K.); matchim.lucrece@gmail.com (L.M.); mark.ndifon@epicentre.msf.org (M.N.); leteki@gwmail.gwu.edu (L.E.);; 4 Tokombere Hospital, Tokombere, Mora BP 74, Cameroon; amtapierre@yahoo.fr; 5 Institut Pasteur, Translational Research Center, Paris Cité University, F-75015 Paris, France; gaelle.noel@gmail.com (G.N.); clauteauxpedro@gmail.com (P.C.); 6 Institut Pasteur, Clinical Research Coordination Center, Paris Cité University, F-75015 Paris, France; anais.perilhou@pasteur.fr; 7 Institut Pasteur, Data Management Core Facility, Paris Cité University, F-75015 Paris, France; marie.sanchez@pasteur.fr; 8 Institut Pasteur de Bangui, Bangui BP 923, Central African Republic; 9 Faculté de Médecine et de Sciences Biomédicales, Yaounde I University, Yaounde BP 1364, Cameroon; 10 Cameroon Society of Epidemiology, Yaounde BP 1411, Cameroon; nkwesch@yahoo.com; 11 Institut Pasteur Medical Center, Paris Cité University, F-75015 Paris, France

**Keywords:** snakebite, envenomation, antivenom, tolerance, sub-Saharan Africa, Cameroon, treatment

## Abstract

Snakebite envenomation (SBE) is a public health issue in sub-Saharan countries. Antivenom is the only etiological treatment. Excellent tolerance is essential in managing SBE successfully. This study aimed to evaluate tolerance of Inoserp^TM^ PAN-AFRICA (IPA). It was conducted on fourteen sites across Cameroon. IPA was administered intravenously and repeated at the same dose every two hours if needed. Early and late tolerance was assessed by the onset of clinical signs within two hours and at a visit two weeks or more after the first IPA administration, respectively. Over 20 months, 447 patients presenting with a snakebite were included. One dose of IPA was administered to 361 patients and repeated at least once in 106 patients. No significant difference was shown between the proportion of adverse events in patients who received IPA (266/361, 73.7%) and those who did not (69/85, 81.2%) (*p* = 0.95). Adverse reactions, probably attributable to IPA, were identified in four (1.1%) patients, including one severe (angioedema) and three mild. All these reactions resolved favorably. None of the serious adverse events observed in twelve patients were attributed to IPA. No signs of late intolerance were observed in 302 patients. Tolerance appears to be satisfactory. The availability of effective and well-tolerated antivenoms would reduce the duration of treatment and prevent most disabilities and/or deaths.

## 1. Introduction

Snakebite envenomation (SBE) is a major public health issue in sub-Saharan Africa (SSA). Recently added to the list of neglected tropical diseases (NTDs) by the World Health Organization (WHO), an SBE prevention and control strategy has been defined to reduce mortality and disability by 2030 [1]. Each year, over 300,000 SBEs are treated in health facilities across SSA, resulting in 10,000 deaths and as many permanent disabilities [2]. However, these figures are underestimated, and the reality is probably more than three times higher [2,3]. SBEs occur in rural areas, in the farming population, which largely explains the general lack of interest in snakebites despite their considerable socioeconomic cost [4,5]. SBE management remains inadequate due to the complex treatment-seeking behaviors of patients, who delay their presentation to hospital, as well as the lack of safe and effective antivenoms [6,7,8]. In addition, most patients struggle to buy more than one or two vials at a time and often wait for a significant worsening to repeat the purchase. In real life, patients have limited access to repeat doses (even when indicated) and, in most cases, even receive an insufficient and inappropriately administered initial dose [2].

In Cameroon, two major families of venomous snakes are responsible for most accidents. The Viperidae, mainly *Echis romani* (formerly *Echis ocellatus*), a potentially lethal species found in savanna, *Bitis*, several species of which are found throughout Cameroon, and *Atheris*, living in central and southern Cameroon, have an enzyme-rich venom that causes inflammation, bleeding disorders, and necrosis. The Elapidae, cobras of the genus *Naja* present throughout Cameroon, and mambas (*Dendroaspis jamesoni*) in the southern forest have a venom composed mainly of toxins causing postsynaptic paralysis, which leads to respiratory arrest, and of phospholipases responsible for necrosis.

The production of an antivenom, the only etiological treatment, is a complex process, which explains the great variations in efficacy and tolerance between brands and even batches from the same antivenom. Their efficacy and safety must be clinically confirmed. Antivenoms are often administered in a peripheral health center that lacks the therapeutic means to manage serious adverse events effectively. High purification of the antibody fragments that make up the majority of current antivenoms has significantly reduced the risk of adverse reactions [4]. Moreover, adverse reactions due to antivenom can be confused with SBE symptoms, additional infection, or stress, which is frequent after snakebites [4]. It is, therefore, essential to look for confounding symptoms before administering the antivenom to avoid incorrect imputations.

Inoserp^TM^ PAN-AFRICA (IPA) manufactured by Inosan Biopharma is widely used in Cameroon and recommended by the Ministry of Public Health. Only IPA, manufactured in Mexico and Spain, was in the process of registration and had special authorization at the time of the study, since 2018. Currently, another antivenom has been in the same situation since 2023 (Panaf-Premium^TM^ manufactured in India). A third antivenom (Equitab^TM^ manufactured in Great Britain) is in the process of being regularized.

The main objective of the ESAA study “Evaluation du Sérum Antivenimeux en Afrique” (=Evaluation of Antivenom in Africa) was to assess the incidence and severity of adverse reactions to IPA under real-life conditions in Cameroon, considering the symptoms present before the antivenom was administered and due to SBE or stress. In addition, we recently published the results of the ESAA study on the IPA’s efficacy [9].

## 2. Results

During the inclusion period, 477 patients presented for snakebite at the 14 study centers. Of these, 3 patients were not included (a child under five, a patient treated with antivenom before presenting to the health center, and a patient with a history of allergy to equine proteins) and 27 patients were excluded for inadequate consent.

The ESAA study enrolled 447 patients. The proportion of men was 51%. The patients’ ages ranged from 5 to 89 years, with a median (interquartile range (IQR)) of 25 (14–40) years (Table 1). Only 11 (2.5%) patients had one or more instances of medical history (four cardiovascular diseases, three rheumatological, two gastrological, one asthma; one HIV infection) 

**Figure 1:**
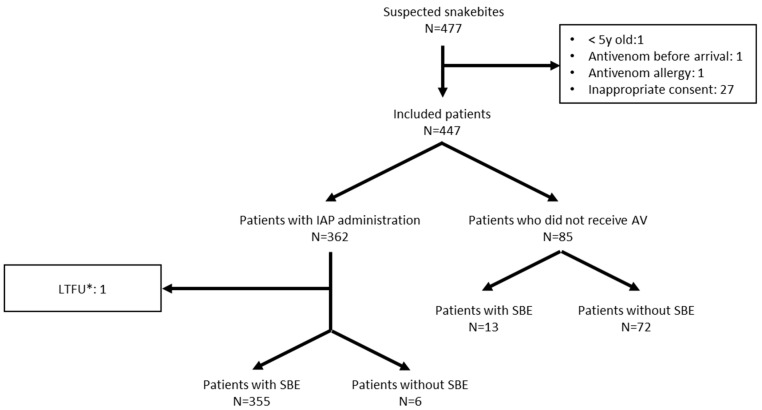
Study flowchart. AV: antivenom; LTFU: lost to follow-up. * Left hospital against medical advice before the post-injection clinical evaluation but was evaluated at long-term visit.

A total of 362 (81%) patients received at least one IPA administration, of whom 106 (29.3%) received multiple administrations (Figure 1). One patient was discharged, against medical advice, after the initial IPA administration but before the clinical evaluation was complete.

Of the 361 patients considered, 355 (98.3%) presented with SBE-related symptoms. The total number of vials administrated was 1016 with a median of 2 [2,3,4] [IQR] vials per patient.

Finally, 85 patients did not receive IPA administration, of whom 72 were not envenomed (Figure 1).

### Tolerance

Of the 361 patients who received IPA and were clinically evaluated, 266 (73.7%) experienced one or more adverse events (Table 2). In 96 (26.6%) patients, these signs occurred within two hours of the initial administration.

Angioedema was reported in one patient (0.3%) within two hours of the initial IPA administration and bronchospasm in another one (0.3%) more than two hours thereafter. Pruritus, urticaria, and diffuse erythema were reported within two hours of IPA administration in four (1.1%), five (1.4%), and one (0.3%) patient, respectively, and more than two hours after administration in four (1.1%), one (0.3%), and one (0.3%) patient, respectively (Table 2). All these events recovered favorably.

Regarding causality assessments, only in four (1.1%) patients were adverse events evaluated as probably linked to IPA administration (Table 3).

In the 85 patients who did not receive IPA, 69 (81.2%) exhibited at least one adverse event. This proportion was not different from the proportion in the 361 patients who received IPA (*p* = 0.95) (Table 2).

Overall, 106 (29.3%) patients received multiple IPA administrations. The proportion of early adverse events decreased significantly (*p* = 0.003) from 96 (26.6%) after the first IPA administration to 16 (15.1%) after the second and to none in the 19 patients who received 3 or more IPA administrations.

Late tolerance (D15) was assessed after a median (IQR) of 16 (15–20) days after enrolment in 302 patients who received IPA. None of them presented symptoms attributable to late IPA intolerance.

SAEs were reported in 12 patients, including 11 deaths—one of them before IPA administration—and 1 death in utero of a fetus in a pregnant woman estimated to be 36 weeks’ amenorrhea (Table 4). None of these SAEs has been attributed to IPA administration.

## 3. Discussion

We conducted the largest study to evaluate antivenom tolerance in Cameroon, a country with a high burden of snakebites.

Early intolerance is related to immediate immunological reactions against heterologous proteins and deleterious substances that may be present in the antivenom (non-immune protein residues, aggregates resulting from protein degradation during improper manufacturing or storage of the product, or preservatives), which can be life-threatening. However, venom injection also corresponds to the penetration of heterologous proteins that determine clinical signs, some of which may be similar to adverse reactions resulting from antivenom administration [4]. The latter were, therefore, systematically sought in patients before the administration of IPA. Moreover, additional infection and stress can cause envenoming-like disorders in the absence of venom and/or symptoms similar to antivenom adverse reactions without inoculation [10,11]. That fact may explain the relatively high number of adverse events reported. However, we attempted to minimize this bias with standardized follow-up, tolerance analysis in patients who did not show symptoms suggestive of intolerance before the administration, and a comparison between proportions of adverse events in patients who did and did not receive IPA.

The imputability of adverse events is based on the circumstances of occurrence, identification of the allergen, and diagnostic means, including biological analysis that was unavailable in this study [4,12,13,14]. The early onset of symptoms less than two hours after contact with the allergen, the symptoms’ characteristics (including anaphylactic shock, angioedema, bronchospasm, respiratory disorders, and skin signs), and rapid disappearance after elimination of the allergen and appropriate treatment are reliable arguments. Once injected, the antivenom takes several days to leave the body, making it impossible to use the criterion of allergen elimination to assess the resolution of intolerance symptoms.

A total of four patients (1.1%) showed evidence of adverse reactions probably attributable to IPA, accounting for five adverse events. Severe signs of intolerance (angioedema and bronchospasm) were observed in two patients (0.6%). All resolved quickly under symptomatic treatment. However, the bronchospasm occurred more than two hours after IPA administration, casting doubt over the imputability of IPA.

Mild adverse events (primarily skin or respiratory signs, including pruritus, urticaria, and dyspnea) were reported within two hours of IPA administration in four patients (1.1%) for pruritus, five (1.4%) for urticaria, one (0.3%) for diffuse erythema, and one for dyspnea (0.3%). Other adverse events occurring within two hours of IPA administration were symptoms not commonly incriminated in allergic responses, consistent with envenomation or stress, or present with the same frequency in subjects who did not receive antivenom.

We did not find a significant difference in the proportions of adverse events in patients who did and did not receive IPA. In this study, we chose to include snakebitten patients with or without envenomation symptoms to follow and collect data at the beginning of their care management at healthcare center level. The fact that patients with signs of envenomation did not receive antivenom reflects the study’s real-life conditions and was mainly linked to under- or over-estimation of local edema intensity. Indeed, the lack of health personnel training and the usual financial barriers to antivenom administration led to its under-administration. The continuing professional development of health personnel and ready availability of antivenoms allowed us to reduce and then eliminate this situation rapidly after the study began. We have chosen to show these results to highlight this situation and better analyze the imputability of adverse events.

The risk of intolerance between patients treated with SDIV and receiving infusions was not analyzed. The circumstances in which both modes of IPA administration were used would have biased the comparison. However, the advantage of SDIV is the rapid bolus administration of the antivenom without the need for rarely available equipment, whereas infusion allows administration of the allergen to be stopped at the onset of symptoms of intolerance. Moreover, given the small number of adverse reactions and the small number of patients presenting a neurotoxic syndrome requiring four vials of antivenom per injection instead of two, we are unable to demonstrate any dose-dependency in the occurrence of adverse reactions. However, the significant decrease in the proportion of early adverse events after re-injection suggests an absence of dose effect.

Late adverse reactions, including serum sickness, occur when the patient has developed antibodies to the antivenom proteins and complexes formed between his/her antibodies and the antivenom precipitate, activating complements and causing an inflammatory reaction [4]. These late adverse reactions occur six to fifteen days after administration of the antivenom and are highly correlated with antivenom dose [15].

In our study, no late adverse events such as serum sickness were identified. However, it was more difficult to estimate the incidence and imputability of IPA. Although a clinical examination of all patients was scheduled fifteen days after the last IPA administration, many visits were delayed or canceled because of logistical constraints (insecurity, difficulty in reaching the patient), especially in northern Cameroon.

Similar results have been reported in several clinical studies with IPA. Chippaux et al. observed 10% adverse reactions in northern Benin and 5.5% in Guinea [16]. Lam et al. reported 4.4% adverse reactions in Senegal [17]. However, symptom collection and IPA imputability criteria were not identical in these different studies. In particular, the evaluators were interested in the possible, not probable, imputability of IPA and did not use Naranjo’s algorithm. 

The low incidence of adverse reactions resulted from the fragmentation of immunoglobulin that removes the complement-binding fragment (Fc), the lyophilization of the product, which ensures that it is well preserved during transport and storage, the absence of preservatives that freeze-drying makes unnecessary [4], and the low protein content of IPA reducing the risk of adverse reactions [12,15].

It is important to note that all patients who experienced adverse reactions recovered quickly after antiallergenic treatment.

## 4. Conclusions

The ESAA study made it possible to evaluate the early tolerance and, to a lesser extent, late tolerance of IPA under real-life conditions in Cameroon.

Tolerance appears to be good with very few severe adverse reactions, none of which were life-threatening, and a low proportion of mild adverse reactions.

IPA (easily stored thanks to freeze-drying) is, therefore, simple to use, with a reduced risk of adverse reactions that are mostly benign, i.e., easy to manage even in isolated health structures.

The training of health personnel in the therapeutic management of envenomation, particularly in rural areas where it is prevalent, is crucial in controlling the burden of SBEs. The availability of effective and well-tolerated antivenoms in peripheral health centers would reduce the time needed for treatment and prevent most disabilities and/or deaths.

## 5. Materials and Methods

### 5.1. Population

The ESAA study was a prospective clinical survey. Patients were recruited between 25 October 2019 and 3 May 2021 in fourteen health centers across Cameroon. The centers were selected by the Ministry of Public Health based on SBE incidence and geographic representativeness to cover a wide range of regions (savannah, forest) with different populations and ecology of snake species.

Inclusion criteria were: (a) snakebite, with or without envenomation, (b) age equal to or greater than five years, (c) absence of known allergy to therapeutic serum of equine origin, (d) no administration of antivenom prior to hospital admission, and (e) signing informed consent.

After inclusion, decisions to initiate antivenom and its dosage were made by the physician–investigator and based on the therapeutic algorithm recommended by the Cameroon Ministry of Health (Appendix A) and the manufacturer’s guidelines.

IPA was provided free of charge to all patients enrolled in the study.

Data were collected in a paper case report form and then entered in a REDCap electronic database [18,19].

Patients were visited at home after hospital discharge to assess clinical progression and observe for signs of adverse reactions, including serum sickness.

### 5.2. Sample Size

The sample size was calculated assuming that the proportion of patients with adverse reactions was 7.5%. To ensure that this proportion did not exceed 10%, with a significance level of 5% and statistical power of 80%, the minimum number of subjects with signs of envenomation to be included was 427. To account for the fact that some patients presenting at the clinical site may be recruited without envenomation (dry or nonvenomous snakebites) and, therefore, would not receive antivenom, it was decided to increase the number of subjects by 10% and include a total of 470 patients who presented with snakebites.

### 5.3. Inoserp™ PAN-AFRICA

Inoserp™ PAN-AFRICA (IPA), currently the reference antivenom in Cameroon, is a lyophilized polyvalent antivenom composed of highly purified fragments of immunoglobulins produced by immunizing horses [20] with the venoms of fourteen species of snakes (*Echis ocellatus*, *E. pyramidum*, *E. leucogaster*, *Bitis gabonica*, *B. nasicornis*, *B. arietans*, *Naja haje*, *N. melanoleuca*, *N. nigricollis*, *N. pallida*, *Dendroapsis polylepis*, *D. viridis*, *D. angusticeps*, and *D. jamesoni*). This antivenom neutralizes the venom of more than eighteen snake species due to its specificity and para-specificity (Appendix B). Each vial contains less than one gram of total protein that neutralizes at least 250 median lethal doses (LD_50_) of *Echis ocellatus*, *Bitis arietans*, *Naja nigricollis*, and *Dendroaspis polylepis* venoms.

Patient management complied with local standards of care and Cameroon Ministry of Public Health recommendations. The mode of IPA administration followed the manufacturer’s recommendations. It was injected either by slow direct intravenous route (SDIV), lasting more than three minutes per 10 mL vial, or by infusion (two 10 mL vials of solution reconstituted and diluted in 50 mL of sterile isotonic saline) over thirty minutes, depending on the severity of symptoms and health personnel practices.

IPA from a single batch (#8IT11001; expiration date November 2021) was used for all patients at all study centers. Vial storage conditions, particularly room temperature, which should not exceed 30 °C (86 °F), were monitored. Exceptions were allowed up to 40 °C (104°F) for a maximum of six months. A storage temperature monitoring system was implemented as part of the study.

Envenomated patients with edema or bleeding, consistent with a viper bite, received two vials (neutralizing 500 LD_50_), whilst those with neurotoxic signs (ptosis, dyspnea, facial or respiratory muscle paralysis) resulting from an elapid bite received four vials (neutralizing 1000 LD_50_). IPA administration was repeated at the same dose two hours after the previous administration in all patients with onset, persistence, or worsening of bleeding or neurotoxic disorders.

### 5.4. Assessment of Tolerance

An adverse event was defined as any undesirable medical event occurring in a participant which was temporally linked to IPA or the study procedures, regardless of cause, and regardless of any potential relationship to IPA or the study procedures. Adverse reaction was defined as any undesirable medical event occurring in a participant and having a causal relationship, whatever its importance (remote, possible, probable, definite), with IPA or the study procedures. Serious adverse event (SAE) was defined as any adverse event or reaction that (a) resulted in death, (b) was life-threatening (patient at risk of death at the time of the event), (c) resulted in temporary or permanent significant disability/incapacity, (d) required hospitalization or prolonged existing hospitalization, (e) led to a congenital anomaly or birth defect, or (f) was medically important (e.g., an event that may not be immediately life-threatening or result in death or hospitalization but could jeopardize the patient’s health or require intervention to prevent one of the other outcomes defined above).

IPA safety and tolerance were assessed in all patients who received at least one IPA administration and had at least one clinical evaluation thereafter. The causality assessment was performed using the Naranjo algorithm [12] (Appendix C Table A1 and Table A2).

Early intolerance was assessed within two hours of IPA administration.

Clinical signs of interest, provided they had not been reported before the IPA administration, were as follows:

- Occurrence of any of the following symptoms: pruritus, urticaria, laryngeal edema, angioedema, bronchospasm, tachycardia/bradycardia, drop in blood pressure (systolic blood pressure < 70 mmHg for children aged five to ten years; <90 mmHg for patients older than ten), anaphylactic shock;
- Onset of fever (axillary temperature ≥ 37.5 °C);
- Digestive disorders (nausea, vomiting, diarrhea, abdominal cramps);
- Agitation, severe headache, or confusion.

Late intolerance was defined as occurrence of any of the following clinical signs more than six days after the first administration of IPA: arthralgia, myalgia, fever (>38°), lymphadenopathy, rash, abdominal pain, splenomegaly, nephritis (defined by the appearance of hematuria and/or proteinuria). In order to assess these outcomes, after hospital discharge, a home visit was scheduled at day 15.

We compared the proportion of adverse events occurring within two hours of IPA administration (early intolerance) and more than two hours thereafter and the proportion of adverse events in patients who did and did not receive IPA.

All SAEs were reported within 24 h to the sponsor, which declared each one to the Ministry of Public Health, the study’s scientific committee, and the IPA manufacturer, which independently assessed the imputability of IPA. The scientific committee was composed of the principal investigators from Paris and Yaoundé, a representative of the sponsor (Institut Pasteur, Paris), and two independent international experts.

### 5.5. Statistical Analysis

The groups were compared using chi-squared tests for categorical variables and nonparametric Wilcoxon or Kruskal–Wallis tests for continuous variables, depending on the number of groups. All analyses were performed using Stata 17 (Stata Corp., College Station, TX, USA).

## Figures and Tables

**Table 1:** Patient’s description.

	Total (N = 447)	Envenomation with AV Injection (N = 356)	Envenomation without AV Injection (N = 13)	No Envenomation with AV Injection (N = 6)	No Envenomation without AV Injection (N = 72)	*p* Value *
**Male, N (%)**	228 (51.0)	182 (51.1)	8 (61.5)	6 (100.0)	32 (44.4)	0.049
**Age (years)**						0.72
Median (IIQ)	25 (14–40)	26 (14–40)	35 (12–49)	30 (21–37)	23 (13.5–35.5)	
Statistical range	5–89	5–87	5–63	20–47	5–89	
**Age group (years)**						0.37
5–11	71 (15.9)	58(16.3)	3 (23.1)	-	10 (13.9)	
12–19	93 (20.81)	70 (19.7)	2 (15.4)	-	21 (29.2)	
>19	283 (63.3)	228 (64.0)	8 (61.5)	6 (100.0)	41 (56.9)	
**Medical history**						0.44
No	436 (97.5%)					
At least one	11 (2.5%)	11 (4.5%)	0	0	0	
**Region**						0.43
Far North	202 (45.2)	161 (45.2)	8 (61.5)	1 (16.7)	32 (44.4)	
North	74 (16.6)	60 (16.9)	1 (7.7)	-	13 (18.1)	
Adamawa	46 (10.3)	38 (10.7)	-	-	8 (11.1)	
Midwest	125 (28.0)	97 (27.3)	4 (30.8)	5 (83.3)	19 (26.4)	
Snake family **						<0.001
Elapidae	10 (2.2)	9 (2.5)	-	-	1 (1.4)	
Viperidae	104 (23.3)	95 (26.7)	1 (7.7)	-	8 (11.1)	
Lamprophiidae	6 (1.3)	3 (0.8)	-	1 (16.7)	2 (2.8)	
Colubridae	19 (4.3)	13 (3.7)	-	-	6 (8.3)	
Nonvenomous	11 (2.5)	3 (0.8)	1 (7.7) §	1 (16.7)	6 (8.3)	
Not identified	297 (66.4)	233 (65.5)	11 (84.6)	4 (66.7)	49 (68.1)	

* chi-squared or Fisher exact tests were used for categorical variables, and Kruskal–Wallis test was used for age; ** as identified by the patient; § identified snake = *Eryx colubrinus* (Boidae family).

**Table 2:** Description of clinical signs in the 361 patients who received at least one IPA and in the 85 patients who did not receive IPA.

	Before Antivenom (N = 361)	After Antivenom Injection * (N = 361)	≤2 h after Initial Antivenom Injection *	>2 h after Initial Antivenom Injection *	Patients without Antivenom (N = 85)	*p* **
Severe signs						
Laryngeal edema	-	-	-	-	-	-
Angioedema	-	1 (0.3)	1 (0.3)	-	-	0.99
Bronchospasm	1 (0.3)	1 (0.3)	-	1 (0.3)	-	0.99
Cough	3 (0.8)	1 (0.3)	-	1 (0.3)	-	0.99
Anaphylactic shock	-	-	-	-	-	-
Cutaneous symptoms						
Pruritus	5 (1.4)	8 (2.2)	4 (1.1)	4 (1.1)	1 (1.2)	0.99
Urticaria	1 (0.3)	6 (1.7)	5 (1.4)	1 (0.3)	-	0.60
Localized erythema	12 (3.3)	2 (0.6)	-	2 (0.6)	2 (2.4)	0.17
Diffuse erythema	1 (0.3)	2 (0.6)	1 (0.3)	1 (0.3)	-	0.99
Respiratory disorders						
Dyspnea	24 (6.6)	3 (0.8)	1 (0.3)	2 (0.6)	3 (3.5)	0.09
Stridor	1 (0.3)	-	-	-	-	-
Cyanosis	1 (0.3)	-	-	-	-	-
Hypoxemia	-	-	-	-	-	-
Digestive disorders						
Nausea	26 (7.2)	8 (2.2)	4 (1.1)	4 (1.1)	1 (1.2)	0.99
Vomiting	34 (9.4)	8 (2.2)	5 (1.4)	3 (0.8)	1 (1.2)	0.99
Diarrhea	9 (2.5)	3 (0.8)	-	3 (0.8)	1 (1.2)	0.57
Abdominal pain	7 (1.9)	7 (1.9)	3 (0.8)	4 (1.1)	-	0.36
Other symptoms						
Fever	66 (18.3)	86 (23.8)	35 (9.7)	51 (14.1)	21 (24.7)	0.86
Blood pressure drop	22 (6.1)	19 (5.3)	10 (2.8)	9 (2.5)	3 (3.5)	0.78
Tachycardia	109 (30.2)	40 (11.1)	18 (5.0)	22 (6.1)	22 (25.9)	**<0.001**
Bradycardia	12 (3.3)	34 (9.4)	10 (2.8)	24 (6.6)	2 (2.4)	**0.031**
Myalgia	129 (35.7)	43 (11.9)	27 (7.5)	16 (4.4)	10 (11.8)	0.97
Malaise	10 (2.8)	1 (0.3)	-	1 (0.3)	-	0.99
Agitation	21 (5.8)	2 (0.6)	1 (0.3)	1 (0.3)	-	0.99
Headache	14 (3.9)	8 (2.2)	4 (1.1)	3 (0.8)	1 (1.2)	0.99
Frisson	6 (1.9)	4 (1.1)	3 (0.8)	1 (0.3)	1 (1.2)	0.99

* clinical signs are considered only if not reported before the first injection of AV. ** comparing the proportion of clinical signs after antivenom injection to the proportion in those who did not receive IPA.

**Table 3:** Causality assessment using the Naranjo algorithm.

	Number of Patients with AE * after AV ^†^ Injection (n = 361)	Unlikely n (%)	Possible n (%)	Probable n (%)	Definite n (%)
Laryngeal edema	-				
Angioedema	1 (0.3)			1 (0.3)	
Bronchospasm	1 (0.3)	1 (0.3)			
Cough	1 (0.3)	1 (0.3)			
Anaphylactic shock	-				
Pruritus	8 (2.2)	1 (0.3)	6 (1.7)	1 (0.3)	
Urticaria	6 (1.7)		4 (1.1)	2 (0.6)	
Localized erythema	2 (0.6)	2 (0.6)			
Diffuse erythema	2 (0.6)		2 (0.6)		
Dyspnea	3 (0.8)	1 (0.3)	2 (0.6)		
Stridor	-				
Cyanosis	-				
Hypoxemia	-				
Nausea	8 (2.2)	4 (1.1)	4 (1.1)		
Vomiting	8 (2.2)	3 (0.8)	5 (1.4)		
Diarrhea	3 (0.8)	3 (0.8)			
Abdominal pain	7 (1.9)	4 (1.1)	3 (0.8)		
Fever	86 (23.8)	51 (14.1)	35 (9.7)		
Blood pressure drop	19 (5.3)	9 (2.5)	10 (2.8)		
Tachycardia	40 (11.1)	22 (6.1)	18 (5.0)		
Bradycardia	34 (9.4)	24 (6.6)	10 (2.8)		
Myalgia	43 (11.9)	16 (4.4)	27 (7.5)		
Malaise	1 (0.3)	1 (0.3)			
Agitation	2 (0.6)	1 (0.3)	1 (0.3)		
Headache	7 (1.9)	3 (0.8)	4 (1.1)		
Frisson	4 (1.1)	1 (0.3)	3 (0.8)		

* adverse event. ^†^ antivenom.

**Table 4:** Clinical description of deceased patients.

ID	Snake	Event	Place of Death	Age	Time between Bite and Hospital Presentation	Time between Bite and Death	IPA Dose	Link with IPA (Score *)	Cause of Death According to Scientific Committee
1	Elapidae	Death	Hospital	6	1 h 30	2 h	0 vial	No (NA **)	Respiratory failure
2	Questionable snake	Death	Hospital	24	5 h	22 h30	6 vials	No (−3)	Digestive hemorrhage or plant poisoning
3	*Echis romani* ^§^	Death	Hospital	12	1 or 2 h	6/7 h	4 vials	No (−3)	Hemorrhagic syndrome + malaria + insufficient antivenom dose
4	*Echis romani* ^&^	Death	Hospital	20	158 h	165 h	4 vials	No (−3)	Severe anemia + brain hemorrhage
5	*Echis romani* ^§^	Death	Home ^†^	45	3 h	168 h	6 vials	No (−3)	Anemia + malaria
6	*Echis romani* ^&^	Death	Home ^†^	41	7 h	120 h	4 vials	No (−3)	Anemia
7	*Naja nigricollis* ^§^	Death	Home ^†^	80	4 h	500 h	2 vials	No (−2)	Heart failure unrelated to envenomation + envenomation
8	*Echis romani* ^&^	Death	Hospital	42	24 h	117 h	4 vials	No (−3)	Cardiovascular collapse
9	*Naja haje* ^§^	Death	Hospital	8	17.4 h	49.6 h	4 vials	No (−3)	Sepsis, inhalation of vomiting, intoxication from traditional treatment, or pulmonary embolism
10	*Echis romani* ^§^	In utero fetal death	Hospital	30	22.9 h	38.5 h	2 vials	No (−2)	Fetal hypotrophy prior to snakebite + hemorrhage
11	*Echis romani* ^§^	Death	Hospital	25	1.7 h	114.3 h	10 vials	No (−3)	Acute renal failure + obstetric hemorrhage due to envenomation
12	*Echis romani* ^§^	Death	Hospital	8	62.5 h	63.5 h	2 vials	No (−2)	Brain hemorrhage

* using the Naranjo algorithm; ** not applicable; ^§^ snake brought by the patient; ^&^ snake identified from the poster; ^†^ discharged against medical advice.

## Data Availability

Data available at DOI: 10.5281/Zenodo.10609046.

## Appendix A. Management Algorithm Recommended by Cameroonian Ministry of Envenomation Patients

**Gradation of edema:**

0. No edema.
1. Localized edema not exceeding the nearest joint.
2. Progressive edema not exceeding 2 contiguous joints.
3. Extensive edema not exceeding the root of the limb.
4. Edema extending beyond the root of the limb (hydrops).

**Gradation of bleeding:**

0. No bleeding.
1. Persistent local bleeding at fang marks for more than one hour.
2. Bleeding from the gums, nose, scars, and recent wounds.
3. Ecchymosis, hematoma, purpura, phlyctens.
4. Internal hemorrhage (peritoneal, meningeal, metrorrhagia, hematemesis, etc.).

**Gradation of neurological disorders:**

0. No neurological disorder.
1. Local anesthesia, tingling affecting the bitten limb.
2. Profuse sweat, saliva and vomiting, miosis.
3. Bilateral ptosis (±speech, vision, hearing and/or swallowing disorders).
4. Respiratory distress, impossibility to communicate.

## Appendix B. List of Snakes for Which the IPA Is Effective (in Bold, Snake Species Present in Cameroon)

**Viperidae:**

- ***Echis ocellatus***;
- *Echis leucogaster*;
- *Echis pyramidum*;
- ***Bitis arietans***;
- *Bitis rhinoceros*;
- ***Bitis nasicornis***;
- ***Bitis gabonica***.

**Elapidae:**

- ***Dendroaspis polylepis***;
- *Dendroaspis viridis*;
- *Dendroaspis angusticeps*;
- ***Dendroaspis jamesoni***;
- ***Naja nigricollis***;
- ***Naja melanoleuca***;
- ***Naja haje***;
- *Naja pallida*;
- *Naja nubiae*;
- *Naja katiensis*;
- *Naja senegalensis*.

## Appendix C. Naranjo Algorithm

**Table A1:** Naranjo algorithm—adverse drug reaction probability scale.

Question	Yes	No	Don’t Know
1. Are there previous conclusion reports on this reaction?	1	0	0
2. Did the adverse event appear after the suspect drug was administered?	2	−1	0
3. Did the AR improve when the drug was discontinued or a specific antagonist was administered?	1	0	0
4. Did the AR reappear when drug was readministered?	2	−1	0
5. Are there alternate causes [other than the drug] that could solely have caused the reaction?	−1	2	0
6. Did the reaction reappear when a placebo was given?	−1	1	0
7. Was the drug detected in the blood [or other fluids] in a concentration known to be toxic?	1	0	0
8. Was the reaction more severe when the dose was increased or less severe when the dose was decreased?	1	0	0
9. Did the patient have a similar reaction to the same or similar drugs in any previous exposure?	1	0	0
10. Was the adverse event confirmed by objective evidence?	1	0	0

**Table A2:** Naranjo Algorithm—interpretation of scores.

Score	Interpretation of Scores
**Total Score ≥ 9**	Definite. The reaction (1) followed a reasonable temporal sequence after a drug or in which a toxic drug level had been established in body fluids or tissues, (2) followed a recognized response to the suspected drug, and (3) was confirmed by improvement on withdrawing the drug and reappeared on re-exposure.
**Total Score 5–8**	Probable. The reaction (1) followed a reasonable temporal sequence after a drug, (2) followed a recognized response to the suspected drug, (3) was confirmed by withdrawal but not by exposure to the drug, and (4) could not be reasonably explained by the known characteristics of the patient’s clinical state.
**Total Score 1–4**	Possible. The reaction (1) followed a temporal sequence after a drug, (2) possibly followed a recognized pattern to the suspected drug, and (3) could be explained by characteristics of the patient’s disease.
**Total Score ≤ 0**	Doubtful. The reaction was likely related to factors other than a drug.
